# A real-world exploration into clinical outcomes of direct oral anticoagulant therapy in people with chronic kidney disease: a large hospital-based study

**DOI:** 10.1007/s40620-024-01930-x

**Published:** 2024-04-02

**Authors:** Ezekwesiri Michael Nwanosike, Hamid A. Merchant, Wendy Sunter, Muhammad Ayub Ansari, Barbara R. Conway, Syed Shahzad Hasan

**Affiliations:** 1 Department of Pharmacy, School of Applied Sciences, University of Huddersfield, Queensgate, Huddersfield, West Yorkshire HD1 3DH UK; 2 Department for Bioscience, School of Health, Sport and Bioscience, The University of East London, London, E16 2RD UK; 3 Calderdale and Huddersfield Pharmacy Services, Anticoagulation Services, Calderdale and Huddersfield NHS Foundation Trust Hospitals, Lindley, Huddersfield, HD3 3EA UK; 4 School of Computing and Engineering, University of Huddersfield, Queensgate, Huddersfield, HD1 3DH UK

**Keywords:** Direct oral anticoagulants (DOACs), Chronic kidney disease, Decision trees, Electronic health records (EHR)

## Abstract

**Background:**

There is limited evidence to support definite clinical outcomes of direct oral anticoagulant (DOAC) therapy in chronic kidney disease (CKD). By identifying the important variables associated with clinical outcomes following DOAC administration in patients in different stages of CKD, this study aims to assess this evidence gap.

**Methods:**

An anonymised dataset comprising 97,413 patients receiving DOAC therapy in a tertiary health setting was systematically extracted from the multidimensional electronic health records and prepared for analysis. Machine learning classifiers were applied to the prepared dataset to select the important features which informed covariate selection in multivariate logistic regression analysis.

**Results:**

For both CKD and non-CKD DOAC users, features such as length of stay, treatment days, and age were ranked highest for relevance to adverse outcomes like death and stroke. Patients with Stage 3a CKD had significantly higher odds of ischaemic stroke (OR 2.45, 95% Cl: 2.10–2.86; *p* = 0.001) and lower odds of all-cause mortality (OR 0.87, 95% Cl: 0.79–0.95; *p* = 0.001) on apixaban therapy. In patients with CKD (Stage 5) receiving apixaban, the odds of death were significantly lowered (OR 0.28, 95% Cl: 0.14–0.58; *p* = 0.001), while the effect on ischaemic stroke was insignificant.

**Conclusions:**

A positive effect of DOAC therapy was observed in advanced CKD. Key factors influencing clinical outcomes following DOAC administration in patients in different stages of CKD were identified. These are crucial for designing more advanced studies to explore safer and more effective DOAC therapy for the population.

**Graphical Abstract:**

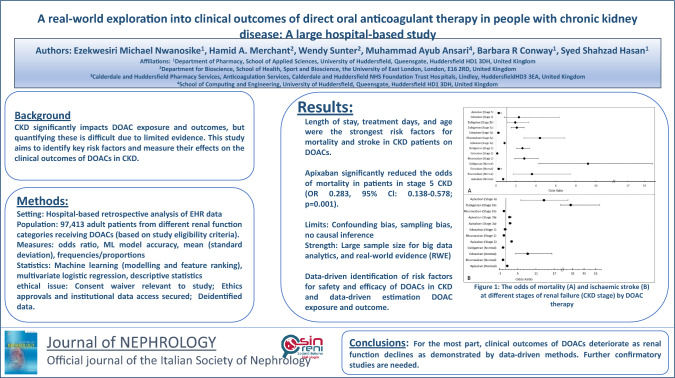

**Supplementary Information:**

The online version contains supplementary material available at 10.1007/s40620-024-01930-x.

## Introduction

Direct Oral Anticoagulants (DOACs) are the first-line anticoagulation therapy prescribed for venous thromboembolism and atrial fibrillation patients with normal kidney function or moderately severe chronic kidney disease (CKD) [1]. The volume of DOAC prescriptions in the National Health Service (NHS) in England has increased steadily given their favourable pharmacokinetic and clinical profile compared to older-generation anticoagulants [2–4]. On the other hand, the incidence of CKD, known to alter the pharmacodynamics and pharmacokinetics of DOACs and to increase the risk of both atrial fibrillation and venous thromboembolism, is also increasing [5, 6].

The fixed-dose regimen of DOACs makes it difficult to tailor DOAC doses for patients with special requirements (e.g., patients with chronic kidney disease who need regular monitoring of kidney function). Nonetheless, to ensure safety and effectiveness in such a cohort of patients, the doses of each drug in the class should ideally be accurately assessed in practice [7]. Useful models for estimating suitable interventions for optimal clinical outcomes (e.g., stroke, death (survival), bleeding, hospitalisations, etc.) would ultimately be driven by high-quality data.

Few studies have highlighted the potential safety concerns (i.e., bleeding, stroke, and death) for DOACs prescribed for patients in different stages of CKD, given the high risk of drug accumulation with deteriorating kidney function [8–11]—for example, patients with CKD on dialysis had twice the risk of stroke and bleeding [12]. These safety concerns are more significant with inappropriate dosing of DOACs in patients in different stages of CKD (hence the need for strict monitoring of kidney function) [13].

Given their dependence on renal clearance to varying extents (apixaban is the least dependent; dabigatran is the most dependent), clinical outcomes of DOACs in patients with CKD have been investigated [14]. This has led to drug regulatory bodies (and relevant clinical governing bodies) establishing the dose adjustment criteria for patients with renal insufficiency for optimising outcomes [15]. However, these criteria are based on either a few controlled studies (e.g., Landmark DOAC trials with creatinine clearance (CrCl) thresholds often ≥ 25 ml/min) with limited data for patients in advanced stages of CKD or limited observational studies with small sample sizes [4]. Food and drug administration (FDA)-approved doses of DOACs in CKD were based on pharmacokinetic modelling since there was a lack of any clinical trial data [11].

The usefulness or accuracy of the dose reduction criteria becomes uncertain for patients with advanced CKD and so warfarin is preferred (extensive clinical data on safety outcomes are available). These gaps in clinical data for DOACs for use in patients with CKD can be filled with electronic health records (EHR)-based observational studies [4]. Advanced statistical methods (e.g., Multivariate Logistic Regression model) can be applied to the curated electronic health records dataset which contains large and richly informative datasets (demographics, intervention, results/outcomes) to optimise safety and effectiveness outcomes associated with the different doses of DOACs in the CKD cohort.

Studies have shown that insights from electronic health records can drastically improve clinical judgement in terms of improving clinical outcomes [12, 16–19]. Admittedly, there is uncertainty regarding the requirements for optimising DOAC therapy in CKD, therefore, this paper aims to identify the most important factors contributing to the clinical outcomes of DOAC therapy in patients in different stages of CKD. This serves as a starting point for further research to explore the safety and efficacy of DOAC therapy in CKD.

## Methods

### Data sources

Data from two hospitals from the CHFT Foundation NHS Trust Hospitals were used in this retrospective observational study. Ethical approval was obtained from the University of Huddersfield Ethics Committee (reference number: SAS-SREIC 21.7.21–7). CHFT granted data access for the study following training and compliance with Information Governance protocols.

Using structured queries on the electronic health records, the hospital's informaticist extracted the feature-rich dataset (reports) that met the study's eligibility criteria. We anonymized and pre-processed (cleaned) the extracted data to ensure it was in an ideal format for analysis. Importantly, patient consent waiver was applied to the study, given that the data from the electronic health records were de-identified and were used for retrospective analysis.

### Study population

We retrospectively identified adult patients between May 1, 2017, and October 20, 2021, both male and female, over the age of 18, who were receiving DOAC therapy. Those who met the inclusion criteria were drawn from a range of CHFT wards (e.g., general medicine, geriatrics, cardiology, and respiratory medicine). They were either admitted directly to these wards or transferred to them. Outpatients and patients admitted to the maternity ward were excluded from the study.

### DOAC therapy

DOAC therapy was prescribed for the management or prevention of ischaemic stroke in atrial fibrillation or the treatment and prevention of venous thromboembolism (deep vein thrombosis or pulmonary embolism), based on local NHS guidelines. Given that many patients had several events (treatment episodes), we chose the last treatment (dose of medication) the patient received (last treatment encounter) to reflect the stable or maintenance dose. Also, we only considered patients who received uniform DOAC therapy throughout; patients whose DOAC therapy was switched were excluded.

### Covariates

For each patient, demographics (e.g., age, gender, ethnicity), clinically relevant variables such as obesity status, height, weight, chronic kidney disease status, bleeding risk, venous thromboembolism risk (using the hospital’s local risk assessment tool), comorbidities, medication (e.g., apixaban, rivaroxaban, edoxaban, and dabigatran), DOAC treatment duration (in days and years), medication dose, and indications, respectively, were extracted from the electronic health records as continuous or categorical features.

The definition of CKD was based on the recent Kidney Disease Improving Global Outcomes (KDIGO) guidelines: *abnormalities of kidney structure or function, present for* > *3 months, with implications for health*. Chronic kidney disease is categorised based on (estimated) glomerular filtration rate (eGFR). The CKD classification standard adopted by the NHS is as follows: stage 1 (normal kidney function) where eGFR ≥ 90 ml/min; stage 2 where eGFR is slightly reduced (60–89 ml/min); stage 3a (eGFR of 45–59 ml/min); stage 3b (eGFR 30–44 ml/min); stage 4 (eGFR 15–29 ml/min) and Stage 5 which depicts kidney failure/end-stage kidney disease (ESKD) (eGFR of 0–15 ml/min).

The Chronic Kidney Disease Epidemiology Collaboration (CKD-EPI) equation adopted by the local NHS standard was used to estimate the renal function of the selected cohort as follows:

eGFR = 141 × min(SCr/κ, 1)α x max(SCr /κ, 1)– 1.209 × 0.993Age × 1.018 [if female] × 1.159 [if Black] (eGFR (estimated glomerular filtration rate) = mL/min/1.73 m2 | SCr (standardized serum creatinine) = mg/dL| κ = 0.7 (females) or 0.9 (males)| *α* = − 0.329 (females) or − 0.411 (males)| min = indicates the minimum of SCr/κ or 1|max = indicates the maximum of SCr/κ or 1| age = years).

The values which are based on CKD staging were encoded accordingly: stage 5 or ESKD (eGFR < 15) = 5; stage 4 (15–29 mL/min) = 4; stage 3b (30–44 mL /min) = 3; stage 3a (45–59 mL/min) = 2; normal kidney function (eGFR ≥ 60) = 1 [9].

### Outcomes

Our study outcomes encompassed; length of stay (in days), all-cause mortality (*deceased*), clinically relevant non-major bleeding in atrial fibrillation and surgical patients, ischaemic stroke, any thromboembolism events, and the number of emergency visits (any hospital emergency visits post-DOAC treatment). We used the International Society on Thrombosis and Haemostasis definition of clinically relevant non-major bleeding, which is ‘any sign or symptom of haemorrhage requiring medical intervention by a healthcare professional or leading to hospitalisation or increased level of care, or prompting a face-to-face evaluation’ [20]

The primary outcomes were clinically relevant non-major bleeding, all-cause mortality, ischaemic stroke, and any thromboembolic events, while secondary outcomes were the length of stay and the number of emergency hospital visits.

### Statistical analyses

The distribution variables extracted for the study were tested for normality using preliminary statistical techniques. Data were summarized using descriptive statistics for continuous (e.g., mean, median, mode, standard deviation) and categorical data (frequency, proportion); intracohort comparison was carried out. Furthermore, possible correlation(s) between the variables were assessed using Pearson’s test. The significance level was set to *p* < 0.05.

The analysis was completed in two phases. In the first phase, machine learning algorithms were implemented and used to identify important features contributing to a specific outcome. In the next phase, multivariate regression models were conducted to examine the association between DOAC therapy and outcomes. The important features identified using machine learning algorithms were entered as confounders in the multivariate regression models. This step provided a strong rationale for selecting relevant covariates in multivariate regression models to examine the statistical associations.

### Machine learning workflow

#### Data cleaning

The most important phase of the machine learning pipeline is data pre-processing. No matter how powerful a machine learning algorithm is, using poor-quality data would yield unrealistic results. Standard data cleaning procedures include removing redundant and irrelevant data, standardising text capitalization (lower case or upper case) and addressing missing values and human errors. A lengthy narrative text was encoded (e.g., the clinical notes in the indication field). The label encoding method was also used to encode categorical features such as gender, race, clinically relevant non-major bleeding/bleeding risk, and stroke/stroke risk. Clinical domain expertise was used to guide feature engineering. Redundant features were removed to reduce the number of features from 49 to 26, and some features were changed to make them more informative.

Data that were missing but had a significant count were labelled as unknown, whereas data that had no significant count (less than 5% of the entire sample) were eliminated. Estimated GFRs > 90 were labelled as 100; for missing values in the body mass index (BMI) column, we replaced them with their computed BMI using the patient’s height and weight. Missing values in the eGFR column were replaced with the average value (imputation of mean). There was a considerable number of human errors in the recording of height and weight. For instance, the height column contained over 12,000 values with incorrect decimal points, leading to numerous outliers. As a result, the data were adjusted, and the BMI was recalculated using the weight and BMI function. Normalizing and scaling variables were further parts of data cleansing.

#### Model development and evaluation

The cleaned data were split into 70% training and 30% test subsets using stratified sampling to ensure the same target class distribution. A range of classification models was trained using the training data and tested on the unseen testing data. Then, the models were evaluated on various performance metrics, including accuracy, precision, recall, F1-score, and confusion matrix—the details on these are shown elsewhere [21]. Figure S1 below summarises the steps of the machine learning pipeline that were implemented.

Besides analysing the dataset using classification models like random forests and decision trees, the same models were used to rank the predictor variables in the overall patient dataset according to the weights of their contribution to the outcomes in the study.

#### Machine learning analysis

Machine learning models are capable of discerning patterns and information from datasets, creating a concise summary of the present data and enabling predictions to be made on new, previously unseen data. The experiments aimed to find the classification model most suitable for the dataset of patients with CKD. Seven (6) well-known machine learning classification models were trained on the cleaned dataset. These selected models employ different algorithms/approaches to learn from data and have different parameters and hyperparameters. The models were trained on the same dataset under the same training and testing settings. The accuracy of the models on the test dataset is shown in Table S1. Apart from the support vector machine, gradient boosting classifier and logistic regression, the remaining models (i.e., random forests and decision trees) achieved excellent accuracy of more than 97%. They learned the patterns in the data better to make acceptable predictions. As a result, they achieved higher values of precision, recall, and F1 scores, as shown in Table S2.

As illustrated in Figures S1 and S2, the decision trees and random forest machine learning algorithms produced a ranking of the features in the dataset based on their strength of influence on clinical outcomes. In descending order, the top 4 features impacting all-cause mortality were treatment days, length of stay, age, and emergency hospital visits.

## Results

### Participants’ characteristics

The number of eligible patients on DOACs extracted from the electronic health records was 97,413, following the adjustment of the number of columns (features) in the dataset. Table 1 gives a statistical summary of the relevant variables. The patients were mostly elderly with an average age of 78.8 years and had at least one comorbidity; a greater proportion had normal kidney function (62.2%)—i.e., non-CKD— and not more than 6% had advanced CKD. The mean eGFR of the patients was 68.5 ml/min.

**Table 1:** Characteristics of the study sample (*n* = 97,413)

Variable	Total patients *n* (%)
Age (yr), Mean ± SD	78.8 ± 11.5
Gender (% Female)	51,127 (52.5)
Ethnicity	
White	90,360 (92.8)
BAME	3056 (3.1)
Other	3997 (4.1)
Height, Mean ± SD	1.6 ± 0.1
Weight, Mean ± SD	74.4 ± 20.8
Medication	
Apixaban	82,073 (84.3)
10 mg BD	2002 (2.1)
5 mg BD	40,924 (42.0)
2.5 mg BD	39,147 (40.2)
Dabigatran	1,125 (1.1)
110 mg BD	807 (0.8)
150 mg BD	282 (0.3)
75 mg BD	36 (0.0)
Edoxaban	366 (0.3)
30 mg OD	243 (0.2)
60 mg OD	123 (0.1)
Rivaroxaban	13,849 (14.2)
10 mg OD	508 (0.5)
15 mg OD	6384 (6.5)
20 mg OD	6957 (7.1)
Treatment days, Mean ± SD	513.9 ± 462.0
Treatment years	
≤ 1 year	48,054 (49.3)
2 years	18,894 (19.4)
3 years	16,316 (16.7)
4 years	10,058 (10.3)
≥ 5 years	4,091 (4.2)
BMI, Mean ± SD	27.2 ± 7.2
eGFR, Mean ± SD	68.5 ± 24.2
eGFR Staging	
Normal kidney function (> 90)	23,726 (24.4)
Non-CKD (Stage 2) (60–89)	36,889 (37.9)
Stage 3a CKD (45–59)	18,397 (18.9)
Stage 3b CKD (30–44)	12,674 (13.0)
Stage 4 CKD (15–29)	5142 (5.3)
Stage 5 CKD (0–14)	585 (0.6)
*Main Indication(s)	
Stroke prophylaxis	80,071 (82.2)
Recurrent VTE prophylaxis	544 (0.6)
VTE treatment	8386 (8.6)
Unclassified	8412 (8.6)
Comorbidity [yes]	82,032 (84.2)
Bleeding risk [yes]	69,886 (71.7)
VTE risk [yes]	96,916 (99.5)

^*^NB: The indications of DOACs overlap across dose regimens

**Fig. 1:**
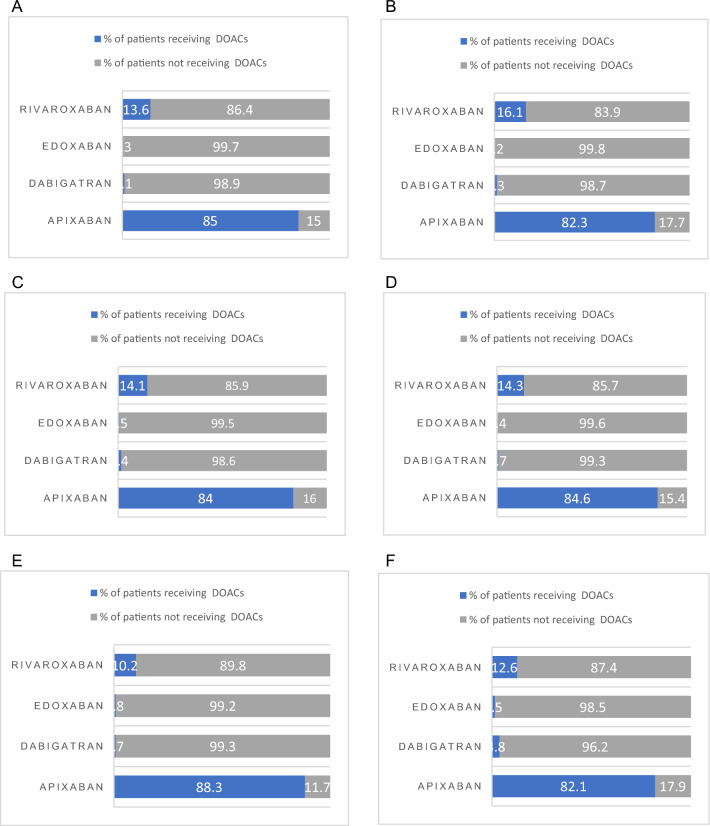
Daily doses of DOACs prescribed by CKD eGFR categories (stages), **A** Non-CKD (Stage 2), **B** Normal kidney function, **C** Stage 3a, **D** Stage 3b, **E** Stage 4 (severe CKD), **F** Stage 5 (renal failure)

The exposure variables were patient demographics, direct oral anticoagulant administered, eGFR, bleeding risk and venous thromboembolism risk, treatment days/years, and comorbidities—these are summarised in Table 1; the outcome variables included length of stay, number of emergency hospital visits, all-cause mortality, clinically relevant non-major bleeding, and ischaemic stroke. Figure 1 summarizes the proportions of individual doses of different DOACs prescribed to patients in different stages of CKD: apixaban (received mostly within the first year of treatment) was the most frequently prescribed agent for all the stages of CKD (including normal eGFR) relative to other DOAC types.

### DOAC therapy and clinical outcomes in patients in different stages of CKD

There was a consistent decrease in mortality with increasing duration of treatment (in years)— per cent mortality peaked in the first year (Table S3). This trend was present in all CKD stages. However, the other clinical outcomes did not improve or deteriorate at a consistent rate throughout the 5-year treatment duration. For instance, the number of emergency hospital visits increased within the first 3 years of treatment in stages 3a, 3b, 4 and 5 of CKD, respectively, before tailing off (Table S4).

Notably, the mean length of stay peaked in the third year of treatment for non-CKD patients and for stage 4 and 5 CKD patients, respectively, whereas, for stage 3a and 3b CKD patients, it peaked in the fourth year of treatment. Also, the highest proportion of patients with stroke was seen in the third year of treatment for non-CKD patients and patients with kidney failure (stage 5 CKD); in the 2nd year for stage 4 CKD, in the 4th year for stage 3a CKD, and in the 5th year for stage 3b CKD, respectively. Very few patients with more severe cases of CKD (or none altogether) had bleeding events, regardless of the treatment duration. The sample size of non-CKD or patients with CKD having thrombotic episodes regardless of the duration of treatment was also low.

The results from the multivariate logistic regression analysis for patients in different stages of CKD regardless of DOAC type are outlined in Table 2. Patients with CKD stage 5 had higher odds of emergency hospital visits than patients with no CKD (OR 1.60, 95% Cl: 1.29–1.98; *p* = 0.001). Compared with patients with no CKD, patients with Stage 4 CKD were associated with lower odds of ischaemic stroke (OR 0.80, 95% Cl: 0.71–0.89; *p* = 0.001) and length of hospital stay (OR 0.79, 95% Cl: 0.71–0.86; *p* = 0.001)—a similar trend was observed in stage 3b, but with higher odds of death (OR 1.61, 95% Cl: 1.49–1.74; *p* = 0.001) and emergency hospital visits (OR 1.95, 95% Cl: 1.80–2.12; *p* = 0.001), respectively—a similar trend was found in stages 3a and 3b. There was evidence of increasing odds of all-cause mortality in patients with CKD compared to patients with no CKD.

**Table 2:** Results of multivariate logistic regression analysis showing the odds of the clinical outcomes for the CKD categories (DOACs)

eGFR Category (CKD staging)	CRNMB		Ischaemic stroke		Any TE events		All-cause mortality		LoS (More than a week)		Emergency visit	
	OR (95% CI)	*p*-value	OR (95% CI)	*p*-value	OR (95% CI)	*p*-value	OR (95% CI)	*p*-value	OR (95% CI)	*p*-value	OR (95% CI)	*p*-value
Normal (> 90)	Referent											
Stage 1 or 2 (60–89)	0.58 (0.49–0.69)	0.001	1.22 (1.17–1.28)	0.001	1.11 (0.94–1.32)	0.223	0.98 (0.95–1.02)	0.366	0.58 (0.55–0.61)	0.001	1.28 (1.23–1.33)	0.001
Stage 3a (45–59.9)	1.58 (1.32–1.90)	0.001	0.97 (0.91–1.02)	0.241	0.76 (0.61–0.95)	0.016	1.10 (1.05–1.15)	0.001	0.71 (0.67–0.76)	0.001	1.51 (1.44–1.58)	0.001
Stage 3b (30–44.9)	0.66 (0.47–0.93)	0.016	0.85 (0.79–0.91)	0.001	1.04 (0.83–1.32)	0.724	1.17 (1.11–1.23)	0.001	0.83 (0.77–0.89)	0.001	1.83 (1.73–1.94)	0.001
Stage 4 (15–29.9)	0.34 (0.14–0.83)	0.018	0.80 (0.71–0.89)	0.001	0.61 (0.40–0.91)	0.016	1.61 (1.49–1.74)	0.001	0.79 (0.71–0.86)	0.001	1.95 (1.80–2.12)	0.001
Stage 5 (< 15)	–	0.991	0.85 (0.64–1.14)	0.276	0.59 (0.22–1.61)	0.301	1.09 (0.89–1.33)	0.424	1.29 (0.97–1.73)	0.084	1.60 (1.29–1.98)	0.001

Variables adjusted for bleeding: ethnic group, gender, length of stay (LoS), BMI Flag, emergency visits, bleeding risk, stroke, mortality, treatment days, comorbidity, apixaban 5 mg, apixaban 2.5 mg

Variables adjusted for stroke: ethnic group, gender, LoS, BMI Flag, emergency visits, bleeding risk, mortality, treatment days, comorbidity, apixaban 5 mg, apixaban 2.5 mg, bleeding, rivaroxaban 15 mg, rivaroxaban 20 mg, thrombosis

Variables adjusted for thromboembolic event: ethnic group, gender, LoS, BMI Flag, emergency visits, bleeding risk, mortality, treatment days, comorbidity, apixaban 5 mg, apixaban 2.5 mg, bleeding, rivaroxaban 15 mg, rivaroxaban 20 mg, stroke

Variables adjusted for mortality: ethnic group, gender, LoS, BMI flag, emergency visits, bleeding risk, treatment days, comorbidity, apixaban 5 mg, apixaban 2.5 mg, bleeding, rivaroxaban 15 mg, rivaroxaban 20 mg, stroke, thrombosis, apixaban 10 mg

Variables adjusted for emergency visits: ethnic group, gender, LoS, BMI, bleeding risk, treatment days, comorbidity, apixaban 5 mg, apixaban 2.5 mg, bleeding, rivaroxaban 15 mg, rivaroxaban 20 mg, stroke, thrombosis, apixaban 10 mg, mortality

Variables adjusted for LoS: ethnic group, gender, BMI flag, bleeding risk, treatment days, comorbidity, apixaban 5 mg, apixaban 2.5 mg, bleeding, rivaroxaban 15 mg, rivaroxaban 20 mg, stroke, thrombosis, apixaban 10 mg, mortality, emergency hospital visits

NB: *P*-value cut-off was 0.001

### Apixaban therapy and clinical outcomes in patients in different stages of CKD

In patients with stage 3a CKD (Table S8), apixaban significantly increased the odds of clinically relevant non-major bleeding (OR 23.68, 95% Cl: 5.84–96.05; *p* = 0.001) and ischaemic stroke (OR 2.45, 95% Cl: 2.10–2.86; *p* = 0.001). On the other hand, there was a significantly lower risk of all-cause mortality (OR 0.87, 95% Cl: 0.79–0.95; p = 0.001) and emergency hospital visits (OR 0.73, 95% CI: 0.66–0.82; *p* = 0.001); a similar trend was observed for ischaemic stroke and emergency hospital visits in stage 3b (Table S9) and stage 4 CKD (Table S10), respectively. Interestingly, Stage 3b CKD was significantly associated with higher odds of prolonged hospital stay (OR 1.24, 95% Cl: 1.08–1.43; *p* = 0.001). Tables S6-S11 present the results of multivariate logistic regression analyses for different stages of CKD.

### Individual DOAC therapy and clinical outcomes in patients in different stages of CKD

The proportion of patients who received apixaban and edoxaban and stayed in hospital for > 1 week increased steadily as the severity of CKD progressed—the proportion of patients that spent less than a week declined. For rivaroxaban and dabigatran, the proportion of patients who spent less than, or more than, one week in hospital, respectively, increased steadily within the first three years of treatment, as the severity of CKD progressed. Also, the proportion of deaths and emergency hospital visits among patients receiving apixaban increased steadily as kidney function declined, within the first four years of treatment. For rivaroxaban, the increase in deaths alone spanned across the 5-year time frame; for edoxaban, a similar trend was observed within the first four years. However, stroke cases (with apixaban) dropped steadily within the five-year time frame; for rivaroxaban, declining cases of stroke were observed within the first four years of treatment.

**Fig. 2:**
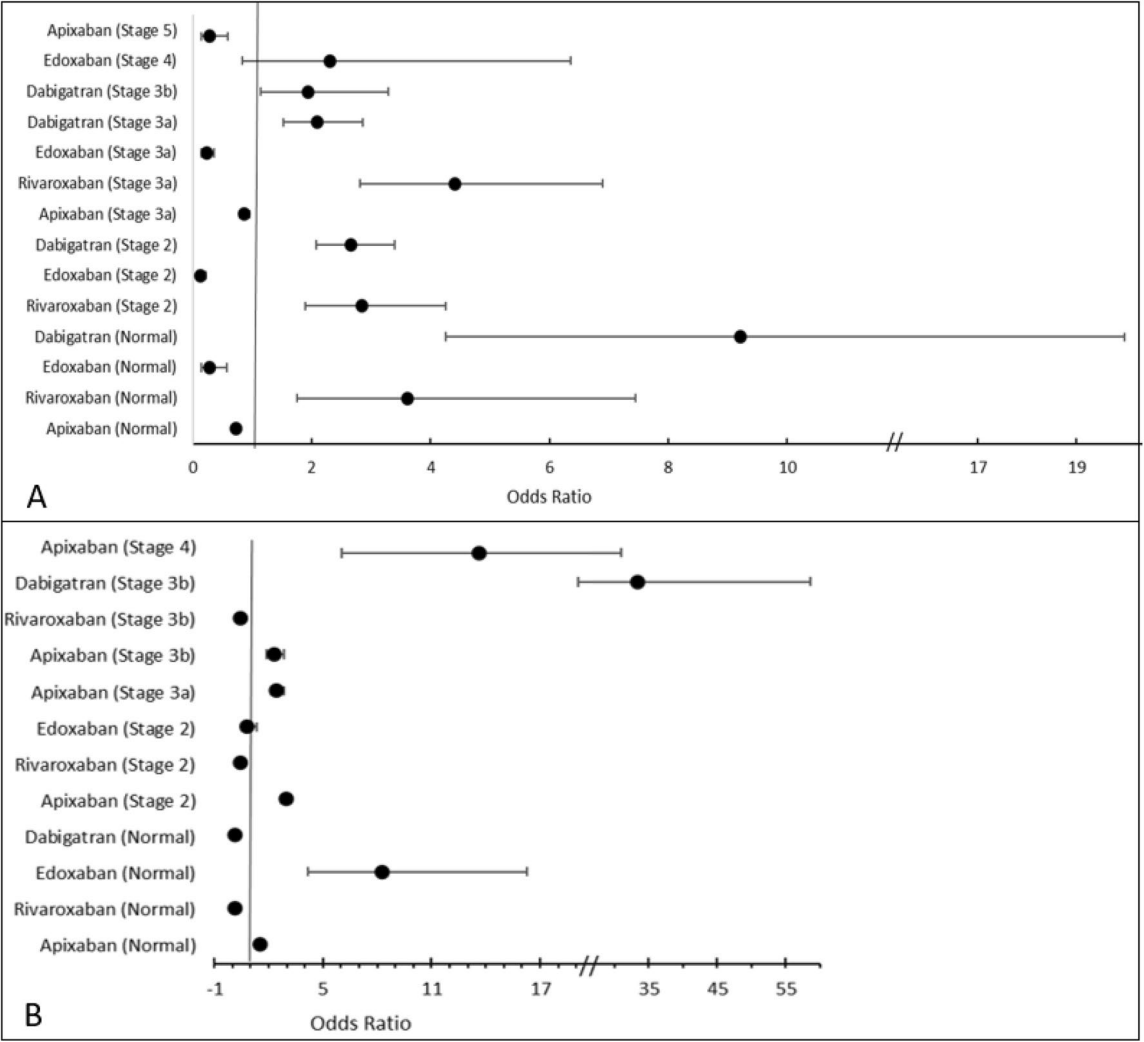
The odds of mortality (**A**) and ischaemic stroke (**B**) at different stages of renal failure (CKD stage) by DOAC therapy

In the case of all-cause mortality, apixaban significantly lowered the odds of death in patients with Stage 5 (OR 0.283, 95% Cl: 0.138–0.578; *p* = 0.001), Stage 3a (OR 0.866, 95% Cl: 0.788–0.953; *p* = 0.001) and no CKD (OR 0.724, 95% Cl: 0.665–0.788; *p* = 0.001) as shown in Fig. 2. Another DOAC that significantly lowered the odds of death in patients with Stage 3a (OR 0.227, 95% Cl: 0.145–0.355; *p* = 0.001), Stage 2 (OR 0.131, 95% Cl: 0.082–0.210; *p* = 0.001) and no CKD (OR 0.277, 95% Cl: 0.134–0.572; *p* = 0.001) was edoxaban. Rivaroxaban significantly reduced the odds of ischaemic stroke in patients with normal kidney function (OR 0.121, 95% Cl: 0.061–0.240; *p* = 0.001), Stage 2 (OR 0.413, 95% CI: 0.253–0.674, *p* = 0.001), and Stage 3b (OR 0.423, 95% CI: 0.344–0.522, *p* = 0.001). However, rivaroxaban significantly increased the odds of death in patients with Stage 2, Stage 3a, and normal kidney function. On the other hand, both apixaban and edoxaban increased the odds of ischaemic stroke.

**Fig. 3:**
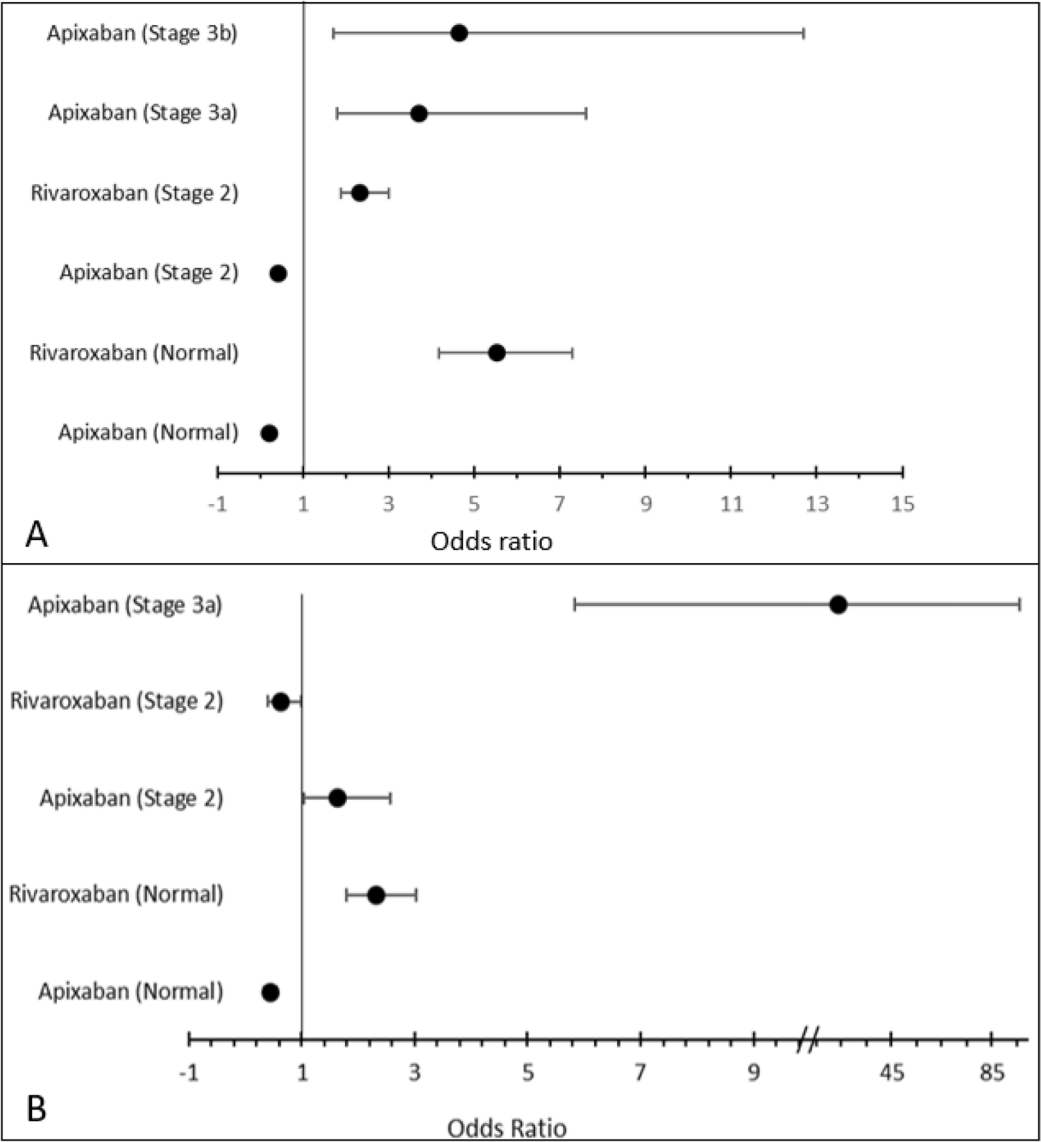
The odds of any thromboembolic events (**A**) and clinically relevant non-major bleeding (**B**) at different stages of renal failure (CKD stage) by DOAC therapy

Figure 3 shows that rivaroxaban significantly increased the odds of thromboembolic events and clinically relevant non-major bleeding in patients with normal kidney function (TE = OR 5.518, 95% Cl: 4.175–7.294; *p* = 0.001|, clinically relevant non-major bleeding = OR 2.329, 95% CI: 1.795–3.022), *p* = 0.001), and CKD stage 2 (TE = 2.336, 95% CI: 1.888–2.991, *p* = 0.001). A majority of patients with Stage 2 CKD and higher received apixaban. Interestingly, apixaban significantly lowered the odds of thromboembolic and clinically relevant non-major bleeding events in patients with normal kidney function and Stage 2, but increased the odds of these events in patients with CKD Stages 3a and 3b.

**Fig. 4:**
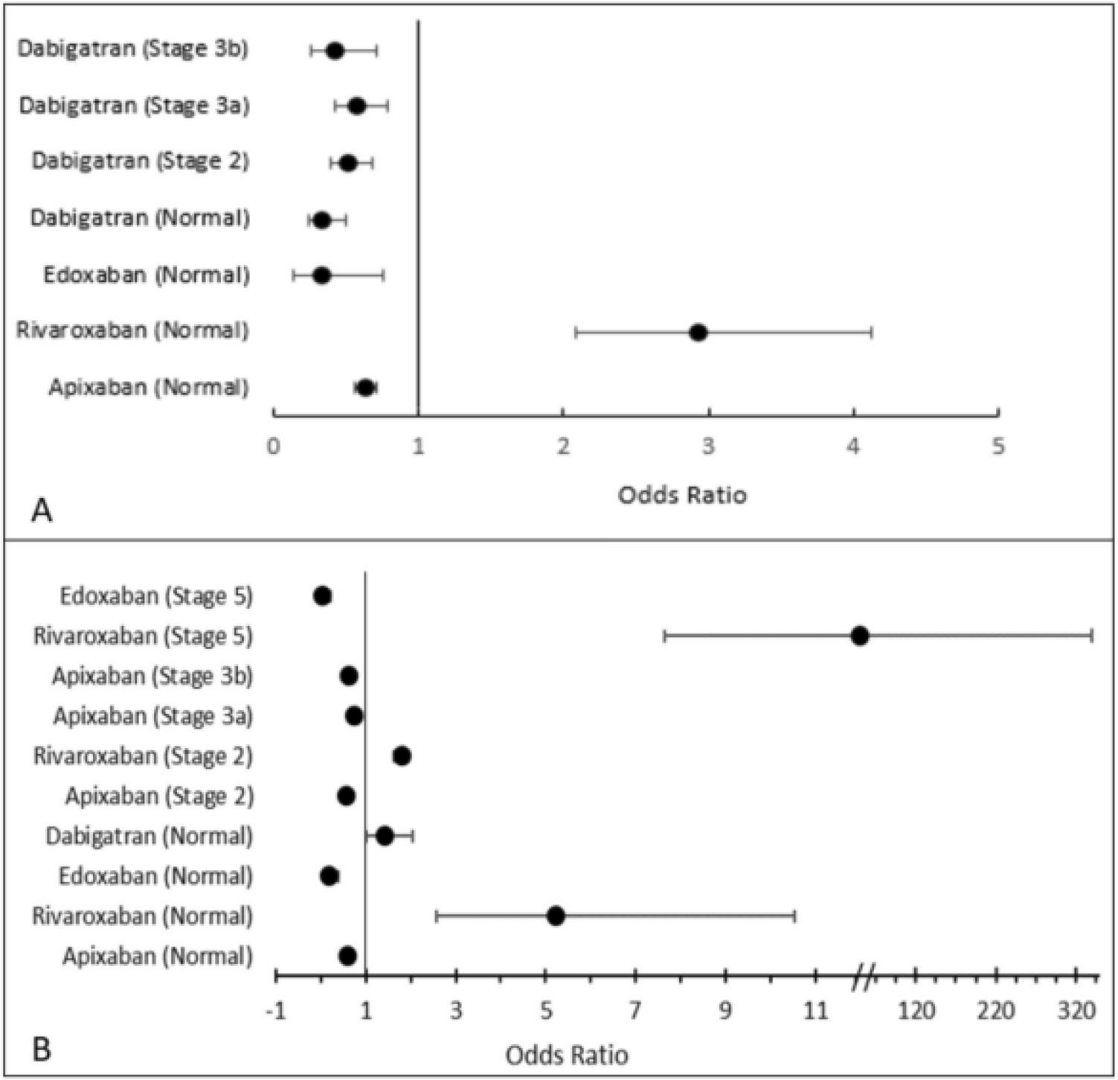
The odds of prolonged hospital stay (**A**) and emergency hospital visits (**B**) at different stages of renal failure (CKD stage) by DOAC therapy

In the case of length of hospital stay, all DOAC regimens, except for rivaroxaban in normal kidney function patients, reduced the odds of prolonged hospital stay, as shown in Fig. 4. In case of emergency hospital visits, rivaroxaban significantly increased the odds of visiting hospital emergency ward(s) in patients with normal kidney function, Stage 2, and Stage 5. Apixaban and edoxaban reduced the odds of emergency hospital visits in patients with normal kidney function, Stage 2, Stage 3a, Stage 3b, and Stage 5 CKD, respectively.

## Discussion

Our study highlights the influence of DOAC type on clinical outcomes at different stages of CKD: results from the analysis of a large real-world dataset provided unique insights that extend the existing clinical evidence. Overall, a partial benefit was derived from the administration of DOACs in patients with kidney function impairment, with some DOAC types being safer or more effective than others.

With DOAC administration (as outlined in the Results), there is increased exposure when renal clearance drops as blood levels of DOACs accumulate to toxic levels. However, the effect of declining kidney function on the exposure of apixaban is less compared to the other DOACs [22]. Reasons for this are the diverse elimination pathways for the drug class of which renal clearance constitutes a minor part. This could explain the lower odds of all-cause mortality and higher odds of ischaemic stroke being significantly associated with apixaban in stage 3a CKD. Ultimately, safety outcomes (i.e., death, bleeding, and emergency admissions) worsen, such as the heightened risk of emergency hospital visits in stage 4 CKD and all-cause mortality and thromboembolic events in stage 3b CKD. These outcomes reflect the pharmacokinetics of DOACs in kidney function impairment. Notably, the ageing patient demographic, comorbid conditions and drug-drug interactions may play an important role in the poor clinical outcomes: ageing is linked to a reduction in GFR; cardiovascular risk factors can accelerate CKD progression and raise the baseline risk of bleeding and thromboembolic complications; co-medication with inhibitors of CYP3A4 enzyme system or p-glycoprotein transporter slows the metabolism (enhances bioaccumulation) [9, 23].

Given that the pharmacokinetics of DOACs in CKD is altered whereas the pharmacodynamics is largely intact [24], improvement in efficacy outcomes (inhibition of blood clotting) with DOAC administration is not unexpected. For instance, there were significantly lower odds of ischaemic stroke in stage 4 CKD (Table 2)— aligning with the decreasing number of cases associated with rivaroxaban in Table S4. Interestingly, the anti-inflammatory and cardiovascular protective effects (e.g., vasodilation and inhibition of platelet aggregation i.e., antiplatelet activity) of DOACs are also contributing factors to better outcomes (efficacy) for venous thromboembolism and stroke prophylaxis in patients in non-advanced stages of CKD [9].

The key features that influenced the outcomes described above were selected and prioritised by the machine learning algorithms—clinically significant factors such as treatment days, emergency hospital visits and length of stay were extensively examined. For instance, emergency hospital visits rose as kidney function declined in patients receiving apixaban, within the first four years of treatment. This may imply that a longer duration of DOAC treatment improved patient survival or that there was no net improvement in treatment outcome with a specific DOAC (switching of therapy may be necessary).

The findings regarding the safety and efficacy of DOACs in CKD patients are inconsistent, and the paucity of relevant data heightens the uncertainty among clinicians when it comes to prescribing DOACs. [23]. Some of the results were similar to those found in published clinical studies, while others differed. For example, Jang et al. [25] showed that dabigatran significantly increased the risk of thromboembolic events in patients in different stages of CKD compared to apixaban. Miao et al. [26] maintained that apixaban and rivaroxaban had similar associations with higher odds of stroke and major bleeding, respectively. Padrini et al. [24] reported that patients receiving apixaban had a higher risk of ischaemic stroke (hazard ratio 4.8; 95% CI 1.30–18.26). It must be noted that, as far as the findings are concerned, the outcomes from trials (with different study designs) were compared indirectly and the sample size of the patients with severe kidney impairment was very small. The finding was supplemented by extrapolated results from pharmacokinetic studies.

On the other hand, Siontis et al. [27] found that apixaban is associated with a significant reduction in major bleeding among atrial fibrillation patients with ESKD (warfarin served as referent): (HR, 0.72; 95% CI, 0.59–0.87; *P* < 0.001), while Arrigoni et al. [18] pointed out that patients on apixaban had a lower risk of thrombotic events. Bonnemeier et al. [28] found that rivaroxaban was associated with a significantly lower risk of ischaemic stroke HR = 0.72; CL = 0.55–0.94; *p* = 0.015, while Chen et al. [17] suggested that rivaroxaban, dabigatran and edoxaban were associated with a significant reduction in the risk of bleeding relative to the referent group HR = 0.76, 95% CI 0.64–0.91, I2 = 62%).

Key ramifications must be considered when interpreting previous studies although they align somewhat with the theory that patients with kidney impairment alongside atrial fibrillation have higher risks of stroke, death, bleeding, and thromboembolic events [12, 18]. Since the Cockroft-Gault Formula was the formula/equation employed in deriving DOAC doses in pivotal trials and is popular in clinical practice, estimation of kidney function based on CKD-EPI would affect the cut-off for dose adjustment leading to potential mis-dosing. However, studies have shown that it gives better GFR estimation for inpatients (ICU patients) whose body weight cannot be measured [29].

Meanwhile, Chan et al. [30] reported slight discordance in dose estimates between Cockroft-Gault and CKD-EPI at eGFR cut-offs of < 15, 15–50, and > 50 mL/min, respectively. The consensus was that CKD-EPI would lead to overdosing, hence worse clinical outcomes compared to the Cockroft-Gault formula. By and large, discrepancies caused by different equations used to estimate DOAC doses in renal patients need to be addressed [25].

Surprisingly, the machine learning algorithms ranked normal kidney function above the severe stages of CKD in terms of importance in determining all-cause mortality. In contrast, the decision tree algorithm assigned higher priority to Stage 3b CKD for predicting stroke. Meanwhile, the decision trees algorithm and random forest showed excellent performance in terms of accuracy in the prediction of stroke and death in patients in different stages of CKD. Other machine learning performance metrics like precision, recall and F1-score yielded similar results. This provides a sound basis (hypothesis) for validating the association between selected risk factors (exposures/interventions) and outcomes.

Comparison of our findings with separate studies in terms of the safety and efficacy of DOACs in CKD is difficult due to variations in study design. However, a common consensus regards poorer safety outcomes with declining kidney function (increasing severity of CKD). There is therefore the need for routine monitoring of DOAC levels in renal patients because the deteriorating kidney function may require dose adjustments if a specific DOAC is still recommended. Medication assessment is also needed to identify concomitant medications interacting with DOACs. By and large, there is a need for prospective clinical trials investigating the impact of DOAC doses in the different stages of CKD, especially severe renal impairment. This would serve as a gold standard for validating the outcomes of our research. It is also pertinent that the therapeutic range of DOACs is established to enable more individualized treatments and optimal outcomes.

The main strength of this study is the large number of patient data that provides a firm basis for generating real-world evidence. This ensures the findings are robust to a large extent and would supplement trial data which has narrower inclusion criteria. Meanwhile, the retrospective nature of the study made it easier to extract relevant data in sufficient amounts. Furthermore, the large dataset is ideal for the application of advanced techniques like regression and machine learning.

The limitations of the study imply that our findings must be interpreted with caution. There was sampling bias as some patient subgroups were underrepresented, hence outcome predictions would not be statistically significant (e.g., an insufficient number of patients with more severe cases of CKD had bleeding events). Also, White patients were overrepresented in the dataset making it difficult to generalise findings to a diverse population, while small sample sizes of some groups or categories make our statistical relationships susceptible to error. In addition, although we adjusted for confounders in our statistical modelling, the observational retrospective design of the study makes it susceptible to confounding biases (e.g. confounding by indication) which can only be considerably ruled out by conducting prospective randomised controlled studies. Furthermore, the CKD staging of the study was based on the chronic CKD-EPI equation rather than the Cockroft-Gault formula used in landmark DOAC trials. This could provide a divergent estimate of the dosing requirements of DOACs. Therefore, there is a need for further confirmatory studies to obtain an accurate eGFR cut-off for optimal DOAC outcomes.

Another drawback is that some characteristics, such as comorbidity, were not entered into the EHR in a systematic or consistent manner that would have allowed for exploratory research. Time-to-event analysis was not feasible due to the retrospective nature or limited scope of the dataset. Since there was such a wide range of comorbidities, the sample sizes for each were small (e.g., diabetes, hypertension, coronary heart disease, cancer, heart failure, CKD, asthma/COPD, osteoarthritis, etc.). As a result, comorbidity was defined in the curated dataset by its presence or absence. Also, there are no set guidelines for choosing the best machine learning model for a particular task when choosing machine learning models. It is standard procedure to test all relevant models and, after thorough model evaluations, choose the one that is most appropriate and accurate. Due to their broad use in pertinent medical situations, decision trees and random forests are recommended over alternative models, such as SCIGAN (eStimating the impacts of Continuous Intervention using GANs) [31].

The study was only based on one NHS trust (CHFT), hence more extensive electronic health record data including numerous NHS trusts are required to produce results that can be more reliably interpreted clinically—this reinforces the need to validate our findings using external datasets. It is crucial to remember that the large percentage of elderly patient groups in the dataset—which is not unique to our study—reflects the UK's ageing population.

## Conclusion

A positive effect of DOAC therapy was observed in advanced CKD. However, clinical outcomes (included in this study) may vary slightly depending on the type of DOAC administered. By and large, the results lend credence to the existing body of evidence on the use of DOACs in different stages of CKD. Ultimately, larger multi-institutional real-world studies as well as prospective clinical trials are crucial for reliably assessing DOAC exposure and clinical outcomes in advanced CKD. This would inform more precise recommendations and identify eGFR cut-offs for optimal DOAC dose levels.

## Supplementary Information

Below is the link to the electronic supplementary material.

Supplementary file1 (DOCX 114 kb)

Supplementary file2 (DOCX 59 kb)

## Data Availability

The datasets generated and/or analysed during the current study are available from the corresponding author upon reasonable request.
