# Supplementary material for: A real-world exploration into clinical outcomes of direct oral anticoagulant therapy in people with chronic kidney disease: a large hospital-based study
Source: J Nephrol. 2024 Apr 2;37(5):1227–40. doi: 10.1007/s40620-024-01930-x (PMC11405428; doi:10.1007/s40620-024-01930-x)
Supplement: Supplementary file 1 — Supplementary file1 (DOCX 114 kb) [file 40620_2024_1930_MOESM1_ESM.docx]

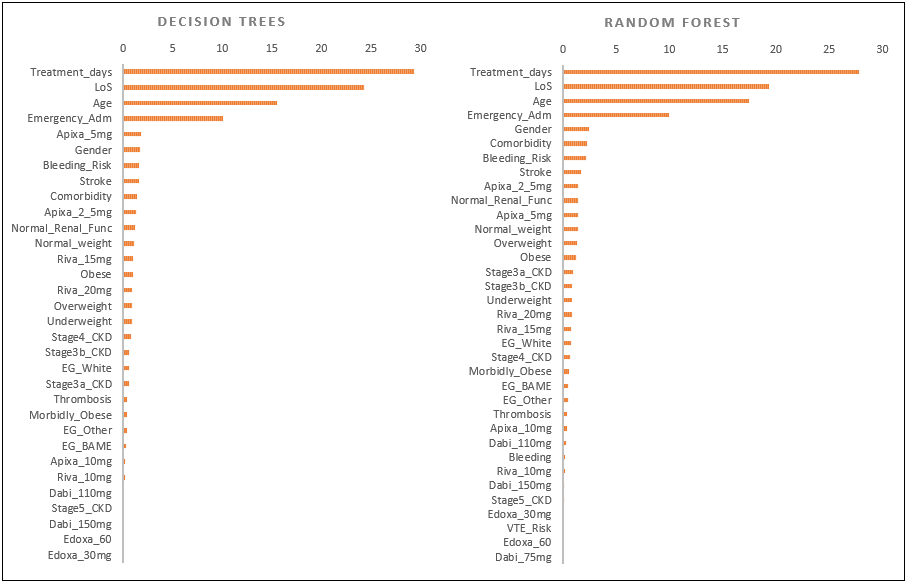


**Figure S1:** Ranking of features with the overall patient dataset according to their importance in contributing to mortality using Random Forest and Decision Trees.

**
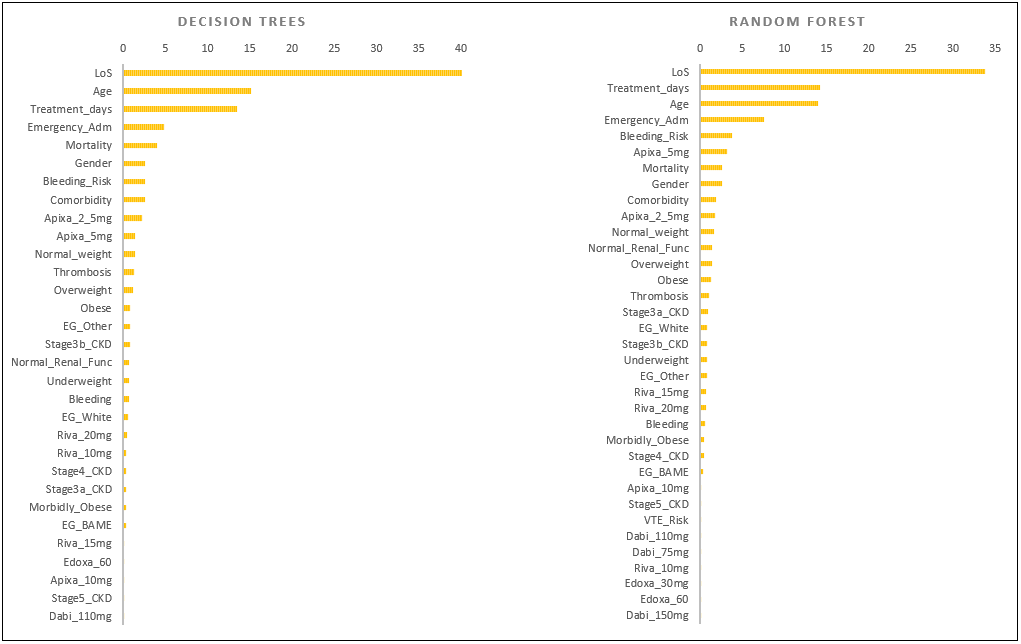
**

**Figure S2:** Ranking of features with the overall patient dataset according to their importance in contributing to stroke using Random Forest and Decision Trees. EG=ethnic group
