# Supplementary material for: A real-world exploration into clinical outcomes of direct oral anticoagulant therapy in people with chronic kidney disease: a large hospital-based study
Source: J Nephrol. 2024 Apr 2;37(5):1227–40. doi: 10.1007/s40620-024-01930-x (PMC11405428; doi:10.1007/s40620-024-01930-x)
Supplement: Supplementary file 2 — Supplementary file2 (DOCX 59 kb) [file 40620_2024_1930_MOESM2_ESM.docx]

**Table S1**: The overall accuracy of each of the ML classifiers used in the study.

| Machine learning algorithm | Accuracy in Stroke prediction | Accuracy in mortality prediction |
| --- | --- | --- |
| Decision Trees | 0.998 | 0.988 |
| k Nearest Neighbour | 0.994 | 0.974 |
| Random Forest | 0.999 | 0.994 |
| Logistic regression | 0.865 | 0.749 |
| Gradient Boosting classifier | 0.911 | 0.811 |
| Support vector machine | 0.911 | 0.811 |

**Table S2**: Performance metrics for the DOAC doses based on different ML models.

| Outcomes | Precision | | | Recall | | | F1-score | | | *Support |
| --- | --- | --- | --- | --- | --- | --- | --- | --- | --- | --- |
|  | DTA | RFA | KNN | DTA | RFA | KNN | DTA | RFA | KNN | DTA, RFA, BAA & KNN |
| Stroke=Yes | 1.00 | 1.00 | 0.98 | 0.99 | 1.00 | 0.98 | 0.99 | 1.00 | 0.98 | 5180 |
| Stroke=No | 1.00 | 1.00 | 1.00 | 1.00 | 1.00 | 1.00 | 1.00 | 1.00 | 1.00 | 24044 |
| Mortality=Yes | 0.99 | 0.99 | 0.98 | 0.99 | 0.99 | 0.97 | 0.99 | 0.99 | 0.97 | 14357 |
| Mortality=No | 0.99 | 0.99 | 0.97 | 0.99 | 1.00 | 0.98 | 0.99 | 0.99 | 0.97 | 14867 |

**Table S3:** Summary of outcomes according to eGFR staging (for all the DOACs)

| eGFR Cat | Treatment Years | LoS in days | Emergency visits | Bleeding Event (CRNMB) | | Ischaemic Stroke | | Any TE event | | All-cause mortality | |
| --- | --- | --- | --- | --- | --- | --- | --- | --- | --- | --- | --- |
|  |  | **Mean (SD)** | **Mean (SD)** | **Yes**  **n (%)** | **No**  **n (%)** | **Yes**  **n (%)** | **No**  **n (%)** | **Yes**  **n (%)** | **No**  **n (%)** | **Yes**  **n (%)** | **No**  **n (%)** |
| Normal Kidney Function (>90) | ≤1yr | 41.04(33.150) | 1.46(1.741) | 80(0.7) | 10957(99.3) | 1936(18.1) | 8741(81.9) | 84(0.8) | 10593(99.2) | 7011(65.7) | 3666(34.3) |
|  | 2yrs | 35.40(33.442) | 3.36(3.289) | 18(0.4) | 4506(99.6) | 633(14.0) | 3891(86.0) | 85(1.9) | 4439(98.1) | 2072(45.8) | 2452(54.2) |
|  | 3yrs | 59.03(67.317) | 3.03(3.739) | 151(3.3) | 4390(96.7) | 1225(27.0) | 3316(73.0) | 10(0.2) | 4531(99.8) | 1134(25.0) | 3407(75.0) |
|  | 4yrs | 37.71(35.152) | 2.77(4.190) | 69(2.4) | 2827(97.6) | 630(21.8) | 2266(78.2) | 50(1.7) | 2846(98.3) | 410(14.2) | 2486(85.8) |
|  | 5yrs | 38.34(47.056) | 1.83(2.703) | 0(0.0) | 1088(100) | 407(37.4) | 681(62.6) | 0(0.0) | 1088(100) | 43(4.0) | 1045(96.0) |
| Non-CKD Stage 2  (60-89) | ≤1yr | 33.91(28.531) | 1.73(2.150) | 114(0.7) | 16574(99.3) | 3004(18) | 13684(82.0) | 161(1.0) | 16527(99.0) | 11020(66.0) | 5668(34.0) |
|  | 2yrs | 29.34(28.182) | 2.88(3.075) | 66(0.9) | 6910(99.1) | 1286(18.4) | 5690(81.6) | 90(1.3) | 6686(98.7) | 3033(43.5) | 3943(56.5) |
|  | 3yrs | 40.12(44.982) | 2.52(2.791) | 47(0.7) | 6691(99.3) | 1923(28.5) | 4815(71.5) | 109(1.6) | 6629(98.4) | 1587(23.6) | 5151(76.4) |
|  | 4yrs | 29.84(34.488) | 2.19(2.634) | 9(0.2) | 4561(99.8) | 1027(22.5) | 3543(77.5) | 47(1.0) | 4523(99.0) | 393(8.6) | 4177(91.4) |
|  | 5yrs | 21.73(28.442) | 1.53(2.347) | 0(0.0) | 1917(100) | 313(16.3) | 1604(83.7) | 18(0.9) | 1899(99.1) | 98(5.1) | 1819(94.9) |
| Stage 3a  (30-39.9) | ≤1yr | 29.22(22.380) | 1.70(1.814) | 146(1.6) | 8950(98.4) | 1292(14.2) | 7804(85.8) | 100(1.1) | 8996(98.9) | 6313(69.4) | 2783(30.6) |
|  | 2yrs | 30.69(24.473) | 2.97(3.220) | 46(1.2) | 3944(98.8) | 649(16.3) | 3341(83.7) | 16(0.4) | 3974(99.6) | 1889(47.3) | 2101(52.7) |
|  | 3yrs | 31.30(27.956) | 3.54(3.374) | 20(0.7) | 2921(99.3) | 443(15.1) | 2498(84.9) | 27(0.9) | 2914(99.1) | 990(33.7) | 1951(66.3) |
|  | 4yrs | 33.79(30.281) | 2.67(2.859) | 0(0.0) | 1634(100.0) | 456(27.9) | 1178(72.1) | 0(0.0) | 1634(100.0) | 190(11.6) | 1444(88.4) |
|  | 5yrs | 21.21(23.260) | 2.54(2.956) | 0(0.0) | 736(100.0) | 56(7.6) | 680(92.4) | 0(0.0) | 736(100.0) | 20(2.7) | 716(97.3) |
| Stage 3b  (≥40) | ≤1yr | 27.17(20.875) | 1.67(1.753) | 40(0.5) | 7306(99.5) | 808(11.0) | 6538(89.0) | 78(1.1) | 7268(98.9) | 1991(72.9) | 5118(27.1) |
|  | 2yrs | 23.68(18.266) | 3.53(3.559) | 0(0.00 | 2670(100.0) | 284(10.6) | 2386(89.4) | 9(0.3) | 2661(99.7) | 1357(50.8) | 1313(49.2) |
|  | 3yrs | 28.97(25.491) | 3.58(2.932) | 0(0.0) | 1652(100.0) | 271(16.4) | 1381(83.6) | 49(3.0) | 1603(97.0) | 595(36.0) | 1057(64.0) |
|  | 4yrs | 30.11(25.036) | 3.49(2.948) | 0(0.0) | 704(100.0) | 79(11.2) | 625(88.8) | 0(0.0) | 704(100.0) | 234(33.2) | 470(66.8) |
|  | 5yrs | 21.70(18.276) | 2.19(2.296) | 0(0.0) | 302(100.0) | 68(22.5) | 234(77.5) | 0(0.0) | 302(100.0) | 15(5.0) | 287(95.0) |
| Stage 4 | ≤1yr | 24.05(19.940) | 1.69(1.475) | 5(0.1) | 3811(99.9) | 235(6.2) | 3581(93.8) | 29(0.6) | 5113(99.4) | 3166(83.0) | 650(17.0) |
|  | 2yrs | 23.08(21.174) | 3.38(2.967} | 0(0.0) | 671(100.0) | 123(18.3) | 548(81.7) | 3(0.4) | 668(99.6) | 313(46.6) | 358(53.4) |
|  | 3yrs | 27.26(23.176) | 3.51(2.846) | 0(0.0) | 404(100.0) | 26(6.4) | 378(93.6) | 3(0.7) | 401(99.3) | 185(45.8) | 219(54.2) |
|  | 4yrs | 21.93(24.055) | 2.74(1.980) | 0(0.0) | 204(100.0) | 21(10.3) | 183(89.7) | 0(0.0) | 204(100.0) | 58(28.4) | 146(71.6) |
|  | 5yrs | 25.04(17.256) | 2.79(2.084) | 0(0.0) | 47(100.0) | 2(4.3) | 45(95.7) | 0(0.0) | 47(100.0) | 0(0.0) | 47(100.0) |
| Stage 5 | ≤1yr | 27.23(15.302) | 1.44(1.305) |  | 431(100.0) | 51(11.8) | 380(88.2) | 0(0.0) | 431(100.0) | 334(77.5) | 97(22.5) |
|  | 2yrs | 28.73(20.656) | 3.54(2.320) |  | 63100.0) | 2(3.2) | 61(96.8) | 4(6.3) | 59(93.7) | 30(47.6) | 33(52.4) |
|  | 3yrs | 51.60(92.006) | 3.60(2.296) |  | 40(100.0) | 7(17.5) | 33(82.5) | 0(0.0) | 40(100.0) | 11(27.5) | 29(72.5) |
|  | 4yrs | 32.22(18.811) | 3.12(3.480) |  | 50(100.0) | 8(16.0) | 42(84.0) | 0(0.0) | 50(100.0) | 2(4.0) | 48(96.0) |
|  | 5yrs | 1.00(—) | — |  | 1(100.0) | 0(0.0) | 1(100.0) | 0.0 | 1(100.0) | 0(0.0) | 1(100.0) |

**Table S4:** Summary of outcomes according to DOAC type in patients at different stages of CKD

| DOACs | CKD Stage | LoS | | Emergency visits | | CRNMB | | Ischaemic Stroke | | Any TE Event | | All-cause mortality | |
| --- | --- | --- | --- | --- | --- | --- | --- | --- | --- | --- | --- | --- | --- |
|  |  | **> 1 week**  **n (%)** | **≤ 1 week**  **n (%)** | **Yes**  **n (%)** | **No**  **n (%)** | **Yes**  **n (%)** | **No**  **n (%)** | **Yes**  **n (%)** | **No**  **n (%)** | **Yes**  **n (%)** | **No**  **n (%)** | **Yes**  **n (%)** | **No**  **n (%)** |
| Apixaban | Normal | 16592(85.0) | 2938(15.0) | 13657(69.9) | 5873(30.1) | 225(1.2) | 19305(98.8) | 4111(21.0) | 15419(79.0) | 101(0.5) | 19429(99.5) | 8715(44.6) | 10815(55.4) |
|  | Non-CKD-2 | 24490(78.1) | 6852(21.9) | 23093(73.7) | 8249(26.3) | 215(0.7) | 31127(99.3) | 7023(22.4) | 24319(77.6) | 303(1.0) | 31039(99.0) | 13578(43.3) | 17764(56.7) |
|  | CKD-3a | 12923(83.6) | 2539(16.4) | 12,145(78.5) | 3,317(21.5) | 210(1.4) | 15252(98.6) | 2686(17.4) | 12776(82.6) | 135(0.9) | 15327(99.1) | 7835(50.7) | 7627(49.3) |
|  | CKD-3b | 9288(86.7) | 1429(13.3) | 8,765(81.8) | 1952(18.2) | 40(0.4) | 10677(99.6) | 1362(12.7) | 9355(87.3) | 132(1.2) | 10585(98.8) | 6365(59.4) | 4352(40.6) |
|  | CKD-4 | 3842(84.6) | 700(15.4) | 3710(81.7) | 832(18.3) | 5(0.1) | 4537(99.9) | 398(8.8) | 4144(91.2) | 24(0.5) | 4518(99.5) | 3296(72.6) | 1246(27.4) |
|  | CKD-5 | 432(90.0) | 48(10.0) | 380(79.2) | 100(20.8) | 0(0.0) | 480(100.0) | 66(13.8) | 414(86.3) | 0(0.0) | 480(100.0) | 298(62.1) | 182(37.9) |
| Rivaroxaban | Normal | 3406(89.0) | 422(11.0) | 3143(82.1) | 685(17.9) | 93(2.4) | 3735(97.6) | 683(17.8) | 3145(82.2) | 128(3.3) | 3700(96.7) | 1715(44.8) | 2113(55.2) |
|  | Non-CKD-2 | 4091(81.6) | 924(18.4) | 4259(84.9) | 756(15.1) | 21(0.4) | 4994(99.6) | 506(10.1) | 4509(89.9) | 122(2.4) | 4893(97.6) | 2230(44.5) | 2785(55.5) |
|  | CKD-3a | 2169(83.7) | 423(16.3) | 2175(83.9) | 417(16.1) | 2(0.1) | 2590(99.9) | 206(7.9) | 2386(92.1) | 8(0.3) | 2584(99.7) | 1379(53.2) | 1213(46.8) |
|  | CKD-3b | 1505(83.0) | 309(17.0) | 1589(87.1) | 234(12.9) | 0(0.0) | 1814(100.0) | 111(6.1) | 1703(93.9) | 4(0.2) | 1810(99.8) | 1091(60.1) | 723(39.9) |
|  | CKD-4 | 444(84.4) | 82(15.6) | 454(86.3) | 72(13.7) | 0(0.0) | 526(100.0) | 6(1.1) | 520(98.9) | 2(0.4) | 524(99.6) | 370(70.3) | 156(29.7) |
|  | CKD-5 | 67(90.5) | 7(9.5) | 64(86.5) | 10(13.5) | 0(0.0) | 74(100.0) | 2(2.7) | 72(97.3) | 0(0.0) | 74(100.0) | 52(70.3) | 22(29.7) |
| Edoxaban | Normal | 44(86.3) | 7(13.7) | 12(23.5) | 39(76.5) | 0(0.0) | 51(100) | 30(58.8) | 21(41.2) | 0(0.0) | 51(100) | 11(21.6) | 40(78.4) |
|  | Non-CKD-2 | 98(82.4) | 21(17.6) | 91(76.5) | 28(23.5) | 0(0.0) | 119(100) | 23(19.3) | 96(80.7) | 0(0.0) | 119(100) | 45(37.8) | 74(62.2) |
|  | CKD-3a | 81(86.2) | 13(13.8) | 86(91.5) | 8(8.5) | 0(0.0) | 94(100.0) | 4(4.3) | 90(95.7) | 0(0.0) | 94(100.0) | 36(38.3) | 58(61.7) |
|  | CKD-3b | 47(87.0) | 7(13.0) | 49(90.7) | 5(9.3) | 0(0.0) | 54(100.0) | 5(9.3) | 5(90.7) | 0(0.0) | 54(100.0) | 34(63.0) | 20(37.0) |
|  | CKD-4 | 38(97.4) | 1(2.6) | 34(87.2) | 5(12.8) | 0(0.0) | 39(100.0) | 0(0.0) | 39(100) | 3(7.7) | 36(92.3) | 34(87.2) | 5(12.8) |
|  | CKD-5 | 9(100.0) | 0(0.0) | 4(44.4) | 5(55.6) | 0(0.0) | 9(100.0) | 0(0.0) | 9(100) | 4(44.4) | 5(55.6) | 5(55.6) | 4(44.4) |
| Dabigatran | Normal | 276(87.1) | 41(12.9) | 279(88.0) | 38(12.0) | 0(0.0) | 317(100) | 7(2.2) | 310(97.8) | 0(0.0) | 317(100) | 229(72.2) | 88(27.8) |
|  | Non-CKD-2 | 328(79.4) | 85(20.6) | 363(87.9( | 50(12.1) | 0(0.0) | 413(100) | 1(0.2) | 412(99.8) | 0(0.0) | 413(100) | 278(67.3) | 135(32.7) |
|  | CKD-3a | 192(77.1) | 57(22.9) | 213(85.5) | 36(14.5) | 0(0.0) | 249(100.0) | 0(0.0) | 249(100) | 0(0.0) | 249(100.0) | 152(61.0) | 97(39.0) |
|  | CKD-3b | 62(69.7) | 27(30.3) | 74(83.1) | 15(16.9) | 0(0.0) | 89(100.0) | 32(36.0) | 57(64.0) | 0(0.0) | 89(100.0) | 66(74.2) | 23(25.8) |
|  | CKD-4 | 31(88.6) | 4(11.4) | 32(91.4) | 3(8.6) | 0(0.0) | 35(100.0) | 3(8.6) | 32(91.4) | 0(0.0) | 35(100.0) | 22(62.9) | 13(37.1) |
|  | CKD-5 | 22(100.0) | 0(0.0) | 100.0 | 0.0 | 0(0.0) | 22(100.0) | 0(0.0) | 22(100) | 0(0.0) | 22(100.0) | 22(100.0) | 0(0.0) |

Table S5: Results of multivariate logistic regression analysis (showing the likelihood of the outcomes (odds ratio) for the CKD categories (Apixaban)

| **eGFR Category (CKD staging)** | **CRNMB** | | **Ischaemic stroke** | | **Any TE events** | | **All-cause mortality** | | **LoS**  (More than a week) | | **Emergency visit** | | |
| --- | --- | --- | --- | --- | --- | --- | --- | --- | --- | --- | --- | --- | --- |
|  | **OR**  **(95% CI)** | **p-value** | **OR**  **(95% CI)** | **p-value** | **OR**  **(95% CI)** | **p-value** | **OR**  **(95% CI)** | **p-value** | **OR**  **(95% CI)** | **p-value** | **OR**  **(95% CI)** | **p-value** |  |
| **Normal**  (>90) | Referent | | | | | | | | | | | | |
| **Non-CKD**  (60-89) | 0.62  (0.51-0.75) | 0.001 | 1.29  (1.23-1.35) | 0.001 | 1.46  (1.16-1.84) | 0.001 | 1.05  (1.01-1.10) | 0.023 | 0.55  (0.52-0.58) | 0.001 | 1.26  (1.21-1.31) | 0.001 |  |
| **Stage 3a**  (45 – 59.9) | 1.57  (1.29-1.91) | 0.001 | 0.91  (0.86-0.97) | 0.003 | 1.37  (1.05-1.78) | 0.021 | 1.20  (1.14-1.26) | 0.001 | 0.69  (0.64-0.74) | 0.001 | 1.44  (1.37-1.52) | 0.001 |  |
| **Stage 3b**  (30 – 44.9) | 0.44  (0.31-0.61) | 0.001 | 0.65  (0.60-0.70) | 0.001 | 2.04  (1.56-2.67) | 0.001 | 1.42  (1.35-1.51) | 0.001 | 0.72  (0.66-0.77) | 0.001 | 1.55  (1.46-1.65) | 0.001 |  |
| **Stage 4**  (15 – 29.9) | 0.11  (0.05-0.28) | 0.001 | 0.56  (0.50-0.62) | 0.001 | 1.00  (0.64-1.58) | 0.995 | 2.12  (1.95-2.30) | 0.001 | 0.52  (0.47-0.57) | 0.001 | 1.55  (1.42-1.68) | 0.001 |  |
| **Stage 5**  (<15) | - | 0.991 | 0.76  (0.57-1.01) | 0.056 | - | 0.993 | 1.17  (0.95-1.45) | 0.149 | 1.01  (0.73-1.38) | 0.973 | 1.35  (1.07-1.69) | 0.010 |  |

NB: P-value cut-off was 0.001

Variables adjusted for stroke: ethnic group, gender, LoS Cat, BMI Cat, emergency visits cat, bleeding risk, mortality, treatment years, comorbidity, bleeding, thrombosis

Variables adjusted for mortality: ethnic group, gender, LoS, BMI Cat, emergency visits, bleeding risk, treatment days, comorbidity, bleeding, stroke, thrombosis

Variables adjusted for emergency visits: ethnic group, gender, LoS, BMI, bleeding risk, treatment days, comorbidity, bleeding, stroke, thrombosis, mortality

Variables adjusted for LoS: ethnic group, gender, BMI flag, bleeding risk, treatment days, comorbidity, bleeding, stroke, thrombosis, mortality, emergency visits

Table S6: Results of multivariate logistic regression analysis (showing the likelihood of the outcomes (odds ratio) for the CKD categories (normal RF)

| **DOACs** | **CRNMB** | | **Ischaemic stroke** | | **Any TE events** | | | **All-cause mortality** | | | **LoS**  (More than a week) | | | **Emergency visit** | | |
| --- | --- | --- | --- | --- | --- | --- | --- | --- | --- | --- | --- | --- | --- | --- | --- | --- |
|  | **OR**  **(95% CI)** | **p-value** | **OR**  **(95% CI)** | **p-value** | **OR**  **(95% CI)** | **p-value** | **OR**  **(95% CI)** | | **p-value** | **OR**  **(95% CI)** | | **p-value** | **OR**  **(95% CI)** | | **p-value** |  |
| Apixaban | 0.436  (0.336-0.565) | 0.001 | 1.527  (1.375-1.697) | 0.001 | 0.205  (0.156-0.271) | 0.001 | 0.724  (0.665-0.788) | | 0.001 | 0.634  (0.564-0.714) | | 0.001 | 0.594  (0.542-0.652) | | 0.001 |  |
| Rivaroxaban | 2.329  (1.795-3.022) | 0.001 | 0.121  (0.061-0.240 | 0.001 | 5.518  (4.175-7.294 | 0.001 | 3.606  (1.747-7.443) | | 0.001 | 2.925  (2.075-4.123) | | 0.001 | 5.216  (2.584-10.528) | | 0.001 |  |
| Edoxaban | NA | - | 8.235  (4.161-16.299) | 0.001 | NA | - | 0.277  (0.134-0.572 | | 0.001 | 0.329  (0.142-758) | | 0.001 | 0.192  (0.095-0.387) | | 0.001 |  |
| Dabigatran | NA | - | 0.111  (0.051-0.240) | 0.001 | NA | - | 9.219  (4.254-19.977) | | 0.001 | 0.344  (0.238-0.499) | | 0.001 | 1.425  (0.999-2.034) | | 0.051 |  |

Variables adjusted for significant bleeding: ethnic group, gender, Cat. length of stay (LoS), BMI Cat, emergency visits, bleeding risk, mortality, treatment years, comorbidity, apixaban, rivaroxaban, edoxaban, dabigatran.

Variables adjusted for stroke: ethnic group, gender, Cat. length of stay (LoS), BMI Cat, emergency visits, bleeding risk, mortality, treatment years, comorbidity, apixaban, rivaroxaban, edoxaban, dabigatran..

Variables adjusted for thromboembolic event: ethnic group, gender, Cat. length of stay (LoS), BMI Cat, emergency visits, bleeding risk, mortality, treatment years, comorbidity, apixaban, rivaroxaban, edoxaban, dabigatran.

Variables adjusted for mortality: ethnic group, gender, Cat. length of stay (LoS), BMI Cat, emergency visits, bleeding risk, stroke, treatment days, comorbidity, apixaban, rivaroxaban, edoxaban, dabigatran.

Variables adjusted for emergency visits: ethnic group, gender, Cat. length of stay (LoS), BMI Cat, emergency visits, bleeding risk, mortality, treatment years, comorbidity, apixaban, rivaroxaban, edoxaban, dabigatran.

Variables adjusted for LoS: ethnic group, gender, BMI Cat, emergency visits, bleeding risk, mortality, treatment years, comorbidity, apixaban, rivaroxaban, edoxaban, dabigatran.

Table S7: Results of multivariate logistic regression analysis (showing the likelihood of the outcomes (odds ratio) for the CKD categories (Stage 2)

| **DOACs** | **CRNMB** | | **Ischaemic stroke** | | **Any TE events** | | **All-cause mortality** | | **LoS**  (More than a week) | | **Emergency visit** | |
| --- | --- | --- | --- | --- | --- | --- | --- | --- | --- | --- | --- | --- |
|  | **OR**  **(95% CI)** | **p-value** | **OR**  **(95% CI)** | **p-value** | **OR**  **(95% CI)** | **p-value** | **OR**  **(95% CI)** | **p-value** | **OR**  **(95% CI)** | **p-value** | **OR**  **(95% CI)** | **p-value** |
| Apixaban | 1.630  (1.036-2.563) | 0.001 | 2.978  (2.692-3.294) | 0.001 | 0.420  (0.338-0.522) | 0.001 |  |  | NA | - | 0.559  (0.516-0.606) | 0.001 |
| Rivaroxaban | 0.627  (0.399-0.987) | 0.044 | 0.413  (0.253-0.674) | 0.001 | 2.336  (1.888-2.991) | 0.001 | 2.840  (1.893-4.261) | 0.001 | NA | - | 1.787  (1.643-1.944) | 0.001 |
| Edoxaban | NA |  | 0.813  (0.503-1.314) | 0.001 | - | 0.996 | 0.131  (0.082-0.210) | 0.001 | NA | - | - | - |
| Dabigatran | NA |  | 0.021  (0.003-0.148) | 0.001 | - | 0.993 | 2.658  (2.076-3.403) | 0.001 | 0.520  (0.396-0.684) | 0.001 | - | - |

Variables adjusted for significant bleeding: ethnic group, gender, Cat. length of stay (LoS), BMI Cat, emergency visits, bleeding risk, mortality, treatment years, comorbidity, ~~apixaban~~, rivaroxaban, edoxaban, dabigatran.

Variables adjusted for stroke: ethnic group, gender, Cat. length of stay (LoS), BMI Cat, emergency visits, bleeding risk, mortality, treatment years, comorbidity, apixaban, rivaroxaban, edoxaban, dabigatran.

Variables adjusted for thromboembolic event: ethnic group, gender, Cat. length of stay (LoS), BMI Cat, emergency visits, bleeding risk, mortality, treatment years, ~~comorbidity, apixaban~~, rivaroxaban, edoxaban, dabigatran.

Variables adjusted for mortality: ethnic group, gender, Cat. length of stay (LoS), BMI Cat, emergency visits, bleeding risk, stroke, treatment days, comorbidity, apixaban, rivaroxaban, edoxaban, dabigatran.

Variables adjusted for emergency visits: ethnic group, gender, Cat. length of stay (LoS), BMI Cat, emergency visits, bleeding risk, mortality, treatment years, comorbidity, apixaban, rivaroxaban, edoxaban, dabigatran.

Variables adjusted for LoS: ethnic group, gender, BMI Cat, emergency visits, bleeding risk, mortality, treatment years, comorbidity, apixaban, rivaroxaban, edoxaban, dabigatran.

Table S8: Results of multivariate logistic regression analysis (showing the likelihood of the outcomes (odds ratio) for the CKD categories (Stage 3a)

| **DOACs** | **CRNMB** | | **Ischaemic stroke** | | **Any TE events** | | | **All-cause mortality** | | **LoS**  (More than a week) | | **Emergency visit** | | |
| --- | --- | --- | --- | --- | --- | --- | --- | --- | --- | --- | --- | --- | --- | --- |
|  | **OR**  **(95% CI)** | **p-value** | **OR**  **(95% CI)** | **p-value** | **OR**  **(95% CI)** | **p-value** | **OR**  **(95% CI)** | | **p-value** | **OR**  **(95% CI)** | **p-value** | **OR**  **(95% CI)** | **p-value** |  |
| Apixaban | 23.676  (5.836-96.050) | 0.001 | 2.453  (2.102-2.862) | 0.001 | 3.711  (1.808-7.614) | 0.001 | 0.866  (0.788-0.953) | | 0.003 | - | - | 0.732  (0.656-0.816) | 0.001 |  |
| Rivaroxaban | - | - | 2.560  (0.917-7.146) | 0.073 | 0.327  (0.160-0.672) | 0.002 | 4.404  (2.814-6.892) | | 0.001 | - | - | 0.719  (0.514-1.006) | 0.054 |  |
| Edoxaban | - | - | 0.391  (0.140-1.090) | 0.073 | - | - | 0.227  (0.145-0.355) | | 0.001 | - | - | 2.070  (0.992-4.319) | 0.053 |  |
| Dabigatran | - | - | - | - | - | - | 2.087  (1.523-2.860) | | 0.001 | 0.571  (0.417-0.784) | 0.001 | - | - |  |

Variables adjusted for significant bleeding: ethnic group, gender, Cat. length of stay (LoS), BMI Cat, emergency visits, bleeding risk, mortality, treatment years, comorbidity, apixaban, rivaroxaban, edoxaban, dabigatran.

Variables adjusted for stroke: ethnic group, gender, Cat. length of stay (LoS), BMI Cat, emergency visits, bleeding risk, mortality, treatment years, comorbidity, apixaban, rivaroxaban, edoxaban, dabigatran..

Variables adjusted for thromboembolic event: ~~ethnic group~~, gender, Cat. length of stay (LoS), BMI Cat, emergency visits, bleeding risk, mortality, treatment years, ~~comorbidity~~, ~~apixaban~~, rivaroxaban, edoxaban, dabigatran.

Variables adjusted for mortality: ethnic group, gender, Cat. length of stay (LoS), BMI Cat, emergency visits, bleeding risk, stroke, treatment days, comorbidity, apixaban, rivaroxaban, edoxaban, dabigatran.

Variables adjusted for emergency visits: ethnic group, gender, Cat. length of stay (LoS), BMI Cat, emergency visits, bleeding risk, mortality, treatment years, comorbidity, apixaban, rivaroxaban, edoxaban, dabigatran.

Variables adjusted for LoS: ethnic group, gender, BMI Cat, emergency visits, bleeding risk, mortality, treatment years, comorbidity, apixaban, rivaroxaban, edoxaban, dabigatran.

Table S9: Results of multivariate logistic regression analysis (showing the likelihood of the outcomes (odds ratio) for the CKD categories (Stage 3b)

| **DOACs** | **CRNMB** | | **Ischaemic stroke** | | **Any TE events** | | **All-cause mortality** | | **LoS**  (More than a week) | | **Emergency visit** | | |
| --- | --- | --- | --- | --- | --- | --- | --- | --- | --- | --- | --- | --- | --- |
|  | **OR**  **(95% CI)** | **p-value** | **OR**  **(95% CI)** | **p-value** | **OR**  **(95% CI)** | **p-value** | **OR**  **(95% CI)** | **p-value** | **OR**  **(95% CI)** | **p-value** | **OR**  **(95% CI)** | **p-value** |  |
| Apixaban | - | - | 2.306  (1.878-2.830) | 0.001 | 4.655  (1.706-12.701) | 0.003 | - |  | 1.240  (1.078-1.426) | 0.003 | 0.623  (0.539-0.719) | 0.001 |  |
| Rivaroxaban | - | - | 0.423  (0.344-0.522) | 0.001 | - |  | - |  | 0.800  (0.695-0.921) | 0.002 | - |  |  |
| Edoxaban | - | - |  |  | - |  | - |  |  |  | - |  |  |
| Dabigatran | - | - | 33.473  (19.128-58.577) | 0.001 | - |  | 1.937  (1.140-3.294) | 0.015 | 0.432  (0.264-0.706) | 0.001 | - |  |  |

Variables adjusted for significant bleeding: ethnic group, gender, Cat. length of stay (LoS), BMI Cat, emergency visits, bleeding risk, mortality, treatment years, comorbidity, apixaban, rivaroxaban, edoxaban, dabigatran.

Variables adjusted for stroke: ethnic group, gender, Cat. length of stay (LoS), BMI Cat, emergency visits, bleeding risk, mortality, treatment years, comorbidity, apixaban, rivaroxaban, edoxaban, dabigatran..

Variables adjusted for thromboembolic event: ethnic group, gender, Cat. length of stay (LoS), BMI Cat, emergency visits, bleeding risk, mortality, treatment years, comorbidity, apixaban, rivaroxaban, edoxaban, dabigatran.

Variables adjusted for mortality: ethnic group, gender, Cat. length of stay (LoS), BMI Cat, emergency visits, bleeding risk, stroke, treatment days, comorbidity, apixaban, rivaroxaban, edoxaban, dabigatran.

Variables adjusted for emergency visits: ethnic group, gender, Cat. length of stay (LoS), BMI Cat, emergency visits, bleeding risk, mortality, treatment years, comorbidity, apixaban, rivaroxaban, edoxaban, dabigatran.

Variables adjusted for LoS: ethnic group, gender, BMI Cat, emergency visits, bleeding risk, mortality, treatment years, comorbidity, apixaban, rivaroxaban, edoxaban, dabigatran.

Table S10: Results of multivariate logistic regression analysis (showing the likelihood of the outcomes (odds ratio) for the CKD categories (Stage 4)

| **DOACs** | **CRNMB** | | **Ischaemic stroke** | | **Any TE events** | | **All-cause mortality** | | **LoS**  (More than a week) | | **Emergency visit** | | |
| --- | --- | --- | --- | --- | --- | --- | --- | --- | --- | --- | --- | --- | --- |
|  | **OR**  **(95% CI)** | **p-value** | **OR**  **(95% CI)** | **p-value** | **OR**  **(95% CI)** | **p-value** | **OR**  **(95% CI)** | **p-value** | **OR**  **(95% CI)** | **p-value** | **OR**  **(95% CI)** | **p-value** |  |
| Apixaban | NA | - | 13.654  (6.005-31.046) | 0.001 | NA | - | NA |  | NA | - | 0.664  (0.510-0.866) | 0.003 |  |
| Rivaroxaban | NA | - | NA | - | 0.001  (0.000-0.011) | 0.001 | NA |  | 0.453  (0.171-1.198) | 0.110 | 0.455  (0.205-1.008) | 0.052 |  |
| Edoxaban | NA | - | NA | - | - | 0.001 | 2.301  (0.833-6.356) | 0.108 | 4.611  (0.627-33.924) | 0.133 | NA | - |  |
| Dabigatran | NA | - | 4.831  (1.040-22.450) | 0.044 |  |  | NA | - | NA | - | NA | - |  |

Variables adjusted for significant bleeding: ethnic group, gender, Cat. length of stay (LoS), BMI Cat, emergency visits, bleeding risk, mortality, treatment years, comorbidity, apixaban, rivaroxaban, edoxaban, dabigatran.

Variables adjusted for stroke: ethnic group, gender, Cat. length of stay (LoS), BMI Cat, emergency visits, bleeding risk, mortality, treatment years, comorbidity, apixaban, rivaroxaban, edoxaban, dabigatran..

Variables adjusted for thromboembolic event: ethnic group, gender, Cat. length of stay (LoS), BMI Cat, emergency visits, bleeding risk, mortality, treatment years, comorbidity, apixaban, rivaroxaban, edoxaban, dabigatran.

Variables adjusted for mortality: ethnic group, gender, Cat. length of stay (LoS), BMI Cat, emergency visits, bleeding risk, stroke, treatment days, comorbidity, apixaban, rivaroxaban, edoxaban, dabigatran.

Variables adjusted for emergency visits: ethnic group, gender, Cat. length of stay (LoS), BMI Cat, emergency visits, bleeding risk, mortality, treatment years, comorbidity, apixaban, rivaroxaban, edoxaban, dabigatran.

Variables adjusted for LoS: ethnic group, gender, BMI Cat, emergency visits, bleeding risk, mortality, treatment years, comorbidity, apixaban, rivaroxaban, edoxaban, dabigatran.

Table S11: Results of multivariate logistic regression analysis (showing the likelihood of the outcomes (odds ratio) for the CKD categories (Stage 5)

| **DOACs** | **Significant bleeding** | | | **Ischaemic stroke** | | **Thromboembolic events** | | **All-cause mortality** | | **LoS**  (More than a week) | | **Emergency visit** | | |
| --- | --- | --- | --- | --- | --- | --- | --- | --- | --- | --- | --- | --- | --- | --- |
|  | **OR**  **(95% CI)** | **p-value** | **OR**  **(95% CI)** | | **p-value** | **OR**  **(95% CI)** | **p-value** | **OR**  **(95% CI)** | **p-value** | **OR**  **(95% CI)** | **p-value** | **OR**  **(95% CI)** | **p-value** |  |
| Apixaban | ~~-~~ | ~~-~~ | 7.340  (1.519-35.472) | | 0.013 | ~~-~~ | ~~-~~ | 0.283  (0.138-0.578) | 0.001 | NA | - | NA | - |  |
| Rivaroxaban | ~~-~~ | ~~-~~ | NA | | ~~-~~ | ~~-~~ | ~~-~~ | NA | - | NA | - | 51.057  (7.662-340.233) | 0.001 |  |
| Edoxaban | ~~-~~ | ~~-~~ | NA | | ~~-~~ | ~~-~~ | ~~-~~ | NA | - | NA | - | 0.034  (0.006-0.200) | 0.001 |  |
| Dabigatran | ~~-~~ | ~~-~~ | NA | | ~~-~~ | ~~-~~ | ~~-~~ | NA | - | NA | - | NA | - |  |

Variables adjusted for significant bleeding: ethnic group, gender, Cat. length of stay (LoS), BMI Cat, emergency visits, bleeding risk, mortality, treatment years, comorbidity, apixaban, rivaroxaban, edoxaban, dabigatran.

Variables adjusted for stroke: ethnic group, gender, Cat. length of stay (LoS), BMI Cat, emergency visits, bleeding risk, mortality, treatment years, comorbidity, apixaban, rivaroxaban, edoxaban, dabigatran..

Variables adjusted for thromboembolic event: ethnic group, gender, Cat. length of stay (LoS), BMI Cat, emergency visits, bleeding risk, mortality, treatment years, comorbidity, apixaban, rivaroxaban, edoxaban, dabigatran.

Variables adjusted for mortality: ethnic group, gender, Cat. length of stay (LoS), BMI Cat, emergency visits, bleeding risk, stroke, treatment days, comorbidity, apixaban, rivaroxaban, edoxaban, dabigatran.

Variables adjusted for emergency visits: ethnic group, gender, Cat. length of stay (LoS), BMI Cat, emergency visits, bleeding risk, mortality, treatment years, comorbidity, apixaban, rivaroxaban, edoxaban, dabigatran.

Variables adjusted for LoS: ethnic group, gender, BMI Cat, emergency visits, bleeding risk, mortality, treatment years, comorbidity, apixaban, rivaroxaban, edoxaban, dabigatran.
